# Genome-Wide Identification of 5-HT Receptor Gene Family in Razor Clam *Sinonovacula constricta* and Their Circadian Rhythm Expression Analysis

**DOI:** 10.3390/ani13203208

**Published:** 2023-10-14

**Authors:** Qiyi You, Qijun Li, Liyuan Lv, Zhihua Lin, Yinghui Dong, Hanhan Yao

**Affiliations:** 1 Key Laboratory of Aquatic Germplasm Resource of Zhejiang, College of Biological & Environmental Sciences, Zhejiang Wanli University, Ningbo 315100, China; z97979y@163.com (Q.Y.);; 2 Ninghai Institute of Mariculture Breeding and Seed Industry, Zhejiang Wanli University, Ningbo 315604, China

**Keywords:** *Sinonovacula constricta*, 5-HT, 5-HT receptor, circadian rhythm

## Abstract

**Simple Summary:**

Serotonin (5-HT) is a highly effective oxytocin widely used in aquaculture. However, the role of 5-HT in mollusks remains unclear. Limited research has been conducted on the structures of 5-HT receptor genes, particularly those associated with ovulatory function. In this study, 5-HT receptors were screened and characterized in the whole genome of *Sinonovacula constricta*. Some 5-HT-binding receptors had been tentatively identified, and their expression pattern exhibited a circadian rhythm. The findings will contribute to a deeper comprehension of the biological role of 5-HT in bivalve ovulation induction and enhance the investigation into its molecular mechanism.

**Abstract:**

Serotonin (5-HT) is primarily distributed in the gastrointestinal and central nervous systems, where it plays a crucial role in regulating various physiological functions such as digestion, reproduction and establishing animal emotions. 5-HT is an effective oxytocin widely used in molluscan aquaculture, and its physiological functions are performed by binding to corresponding 5-HT receptors (5-HTRs). In this study, seven *5-HTR* genes of *Sinonovacula constricta* (*Sc5-HTRs*) were identified and analyzed, and they were designated as *Sc5-HT1A*, *Sc5-HT1D*, *Sc5-HT2-1*, *Sc5-HT2-2*, *Sc5-HT2-3*, *Sc5-HT4* and *Sc5-HT6*. Phylogenetic analysis showed that the seven *Sc5-HTRs* were conserved among mollusks, and the Sc5-HTRs were all transmembrane proteins. The seven *Sc5-HTR* genes were distributed on chromosome 1, 2, 13 and 14. After injecting 5-HT, there was a significant increase in mRNA expression levels of *Sc5-HT1A* (*p* < 0.05) and *Sc5-HT2-3* (*p* < 0.01), while *Sc5-HT4* decreased significantly (*p* < 0.01) compared to control groups which might be effective 5-HT receptors. Furthermore, two of the receptors (*Sc5-HT2-3* and *Sc5-HT4*) were expressed in the circadian rhythm patterns, indicating their potential influence on the nocturnal spawning of *S. constricta*. Overall, these findings provide a theoretical basis for understanding the structures and functions of *5-HTR* gene family members, and may facilitate the artificial propagation of mollusks.

## 1. Introduction

5-hydroxytryptamine (5-HT), or serotonin, a kind of canonical neurotransmitter, can increase a delighted atmosphere and is therefore called “the hormone of happiness” along with dopamine and norepinephrine [1]. As neurotransmitters, neuromodulators and neurohormones, and ancient signaling molecules, 5-HT widely exists in the plant and animal kingdoms [2,3] and plays important roles in mood, appetite, sleep, sex and temperature regulation [4], which are required to be combined with specific 5-HT receptors (5-HTRs) located on the surface of cell membrane. Up to now, seventeen types of 5-HTRs, classified into seven families (5-HT1~5-HT7), have been discovered in vertebrates, of which six members (5-HT1, 5-HT2, 5-HT4, 5-HT5, 5-HT6 and 5-HT7) are G-protein coupled receptors (GPCRs), and one member (5-HT3) is an ionotropic receptor [5]. Additionally, invertebrates have been reported to possess five 5-HTR families (5-HT1, 5-HT2, 5-HT4, 5-HT6, 5-HT7) [6,7,8,9,10]. In invertebrates there are three ascertained 5-HTR families (5-HT1, 5-HT2, 5-HT7), which are orthologous to mammals. At present, 5-HT and its receptors have been identified in nematodes [11,12], flatworms [3], insects [13,14], mollusks [15,16] and so on. In flatworms, 5-HT7 was highly expressed in the muscle and nervous systems, which provided evidence for the subsequent research on neurodevelopment [1]. Due to the distinct functions of the various 5-HTRs, most studies have been focused on single receptors of 5-HT. Receptor antagonists or agonists with specific binding affinity are the fundamental tools for identifying distinct subtypes of 5-HTRs in vertebrates. Nevertheless, identifications of 5-HTRs by pharmacological experiments were not satisfactory in invertebrates [17], hence the efficient approach for 5-HTR identification in invertebrates requires exploring.

Moreover, 5-HT could modulate spawning, and induce gonadal maturation and meiosis reinitiation of prophase-arrested oocytes in mollusks [18,19,20,21], which are required to be combined with specific 5-HT receptors (5-HTRs) located on the surface of cell membrane. In bivalves, 5-HT promotes oocyte maturation, sperm motility and sequential spawning [22], making it an effective oxytocin for spawning. For example, 5-HT could induce spawning in the blue mussel *Mytilus edulis*, and the effective receptor has been proved to be 5-HT2 [23]. During the peak breeding season of Pacific abalone *Haliotis discus hannai*, the mRNA expression level of *Hh5-HTR* increased significantly [16].

The razor clam *Sinonovacula constricta* is an economically important bivalve with fast growth, high yield and a short production cycle, and artificial cultivation has been developed in the last two decades. Compared to other mollusks, the spawning time for razor clams is usually at night [24,25], and injection of 5-HT could induce spawning, indicating the important role of 5-HT in this process through binding to corresponding 5-HTRs. To elucidate the specific types of 5-HTRs that bind during 5-HT-induced spawning, *Sc5-HTRs* were identified by bioinformatic analysis based on the *S. constricta* genome, and the mRNA expression levels of *Sc5-HTRs* after injecting 5-HT were investigated. Furthermore, in order to explore the relationships between 5-HT and nocturnal spawning, the expression patterns of *Sc5-HTRs* were analyzed within 72 h. In summary, these findings provide a theoretical foundation for comprehending the structures and functions of 5-HTR gene family members in mollusks, which could facilitate the cultivation of mollusks through artificial breeding techniques. 

## 2. Materials and Methods

### 2.1. Experimental Animals and Sample Collections

The razor clams with mature gonads full of sperms or eggs (average shell length of 64.40 ± 2.81 mm and body weight of 14.29 ± 2.17 g), were collected from a commercial shellfish farm in Ningbo, Zhejiang province, and then were maintained in aerated seawater (temperature 24 ± 1.0 °C, salinity 20 ± 1 ppt) under a light cycle of 12 L:12 D for 7 days. The artificial lights were turned on (the light intensity 359 ± 20 lx), and the light shone directly into the tank from 08:00 to 20:00 to simulate the daytime (12 h light, 08:00–20:00), and black cloths covered the tank from 20:00 to 08:00 to simulate the nighttime (12 h dark, 20:00–08:00) [24]. The microalgae *Chlorella vulgaris* was fed twice a day at 6:00 and 18:00, and the culture water was continuously aerated and changed once a day. Eight individuals (four males and four females) were randomly selected at four time points (00:00, 06:00, 12:00 and 18:00) per day over a period of three days (72 h), tissues including foot, siphon, mantle, gill, hepatopancreas, adductor muscle and male and female gonads were dissected, immediately frozen in liquid nitrogen and stored at −80 °C for tissue expression analysis of *Sc5-HTRs*. 

### 2.2. Identification and Sequence Analysis of Sc5-HTRs

First of all, the 17 5-HT receptor protein sequences in humans (HTR1A: NP_000515.2; HTR1B: NP_000854.1; HTR1D: NP_000855.1; HTR1E: NP_000856.1; HTR1F: NP_000857.1; HTR2A: AAM21129.1; HTR2B: AAN01277.1; HTR2C: AAM21130.1; HTR3A: AAM21131.1; HTR3B: KAI4074216.1; HTR3C: KAI4032763.1; HTR3D: KAI4032762.1; HTR3E: KAI4032769.1; HTR4: KAI4023363.1; HTR5A: NP_076917.1; HTR6: NP_000862.1; HTR7: KAI4076793.1) downloaded from NCBI were used as the query sequences to perform local Blastp screening in BioEdit for similar sequences in the genome of *S. constricta* (WSYO00000000.1), and the E-value was set at 1 × 10^−10^. NCBI CD-search (https://www.ncbi.nlm.nih.gov/Structure/cdd/wrpsb.cgi, accessed on 20 December 2021) was used to search the conserved domains of Sc5-HTRs. 

The physicochemical properties of Sc5-HTRs, including protein length, molecular weight (MW) and isoelectric point (pI) were predicted by ProtParam (https://web.expasy.org/protparam/, accessed on 20 April 2022) and Protscale (https://web.expasy.org/protscale/, accessed on 20 April 2022), respectively. TMHMM online software (https://services.healthtech.dtu.dk/service.php?TMHMM-2.0, accessed on 12 May 2022) was used to predict protein transmembrance structures, ProtComp online software (http://linux1.softberry.com/, accessed on 12 May 2022) was used to predict subcellular localizations and phosphorylation site statistics for corresponding sequences were analyzed by DTU online software (https://services.healthtech.dtu.dk/service.php?NetPhos-3.1, accessed on 12 May 2022). 

### 2.3. Multiple Sequence Alignment and Phylogenetic Analysis

The multiple sequence alignments and transmembrane structure comparison of the protein sequence of Sc5-HTRs were accomplished through the DNAMAN 6.0.3.99 software. The 5-HTRs’ protein sequences in model animals and other mollusks were downloaded from NCBI, and GenBank numbers are listed in Supplementary Table S1. The phylogenetic tree of 5-HTRs was established using MEGA 7 with the maximum likelihood (ML) method (3000 bootstrap replicates). 

### 2.4. Motif Patterns and Chromosome Location

The obtained Sc5-HTR protein sequences were submitted to MEME (Multiple Expectation Maximization for Motif Elicitation, http://alternate.meme-suite.org/, accessed on 20 May 2022) for identification of conserved motifs, and the distributions of *Sc5-HTRs* on *S. constricta* chromosomes were visualized using TBtools [26]. 

### 2.5. Prediction of Tertiary Structure of Sc5-HTR Proteins

The tertiary structures were constructed by Phyre2 (http://www.sbg.bio.ic.ac.uk/phyre2/html/page.cgi?id=index, accessed on 20 May 2022), and then were visualized using the PyMOL software (version: 3.8.2).

### 2.6. Preliminary Identification of Effective Sc5-HTRs That Bind to 5-HT

The mature razor clams, with an average shell length of 64.40 ± 2.81 mm and body weight 14.29 ± 2.17 g, were selected and divided into two groups (EG: experimental group, CG: control group) to identify effective Sc5-HTRs that bind to 5-HT. 5-HT (solution serotonin creatinine sulfate salt monohydrate, Baichuan, Ningbo, China) was formulated at 10^−4^ mol/L in 300 mL of filtered natural seawater. Subsequently, 300 μL 5-HT (EG group) or 300 μL sterilized seawater (CG group) was injected into the mature gonads of the clams in each group by microinjector at 18:00. Two hours after injection (at 20:00), most of razor clams (75% on average) in the EG group had spawned, while only 32% razor clams spawned in the CG group. Tissues including foot, siphon, mantle, gill, hepatopancreas, adductor muscle and male and female gonads were collected from ten clams from each group, frozen in liquid nitrogen and then stored at −80 °C until total RNA extraction.

### 2.7. Circadian Rhythm Expression Patterns of Sc5-HTRs

In order to investigate the correlation between *Sc5-HTRs* and nocturnal spawning of the razor clams, the circadian rhythm expression analysis of *Sc5-HTRs* was conducted. Mature razor clams (average shell length = 64.40 ± 2.81 mm, average body weight = 14.29 ± 2.17 g) were randomly placed into three tanks under the same experimental conditions. The microalgae *Chlorella vulgaris* was fed twice a day at 6:00 and 18:00, and the culture water was continuously aerated and changed once a day. Considering the pre-experiment and nocturnal spawning of *S. constricta* [24], the samples were collected at four time points (00:00, 06:00, 12:00 and 18:00) per day within 72 h. Each time corresponded to three parallel tanks. Six males and six females were randomly selected at each time (two clams from each tank), and tissues (foot, siphon, mantle, gill, hepatopancreas, adductor muscle, ovary and testis) were dissected and stored at –80 °C to detect gene expressions.

### 2.8. RNA Extraction and qRT-PCR

Total RNA of tissues was extracted with Trizol (Vazyme, Nanjing, China), and RNA quality and quantity were evaluated via 1.5% agarose gel electrophoresis and NanoDrop system (Nano-300, Hangzhou, China), respectively. Then cDNA was synthesized with a Prime-Script^TM^ RT reagent kit (TaKaRa, Tokyo, Japan) according to the manufacturer’s instruction. Finally, the expression pattern of *Sc5-HTRs* was examined by ChamQTM Universal SYBR^®^ qPCR Master Mix (Vazyme, Nanjing, China) in LightCycler^®^ 480II (Roche, Indianapolis, IN, USA). The primers used in the experiment are shown in Table 1, and the *RS9* gene was selected as an internal reference gene. The PCR amplification system was as follows: 8 μL of cDNA (different genes choose appropriate dilution times), 1 μL forward primer, 1 μL reverse primer and 10 μL of SYBR qPCR Master Mix. The reaction procedure was 95 °C for 30 s, followed by 40 cycles of denaturation at 95 °C for 15 s and annealing at 60 °C for 1 min. All samples were performed with four biological parallels and three technical replicates. The expression levels of *Sc5-HTRs* were calculated using the method of 2^−ΔΔCT^, and then were processed in GraphPad Prism 8.0.

### 2.9. Statistical Analysis

Statistical analyses of the data were performed using SPSS 26.0 (IBM Crop, Armonk, NY, USA). The method of 2^−ΔΔct^ was used to analyze the expression level of *Sc5-HTRs*, and the results were presented as mean ± S.E. The data were assessed by one-way analysis of variance (one-way ANOVA), and Student’s *t*-test was performed on each data set. Differences were considered significant if *p* < 0.05, and *p* < 0.01 denoted an extremely significant difference.

## 3. Results

### 3.1. Sequence Analysis of Sc5-HTRs

Seven *Sc5-HTRs* were retrieved from the *S. constricta* genome, and the results of the similarity analysis showed that members of the *Sc5-HTR* family exhibited higher homology with other species and were most similar to the hard clam *Mercenaria mercenaria* (Supplementary Tables S1, Table 2). Neither sequence alignment nor phylogenetic analysis could determine the subtype due to the uniqueness of *Sc5-HT2*, which contained three members and was named by numbers rather than letters. Thus, the seven *Sc5-HTRs* were named *Sc5-HT1A*, *Sc5-HT1D*, *Sc5-HT2-1*, *Sc5-HT2-2*, *Sc5-HT2-3*, *Sc5-HT4* and *Sc5-HT6*, respectively.

The fundamental physicochemical properties of *Sc5-HTR* genes are shown in Table 3. In general, the molecular weights of the Sc5-HTRs ranged from 43.945 to 60.478 kDa, and theoretical pI values ranged from 7.88 to 9.40, respectively. All of the Sc5-HTRs were subcellularly localized to the cell membrane, which was the typical characteristics of GPCRs. Interestingly, all of the Sc5-HTRs (except Sc5-HT2) possessed 28~50 predicted phosphorylation sites, while Sc5-HT2-1, Sc5-HT2-2 and Sc5-HT2-3 contained over 60 potential phosphorylation sites. 

### 3.2. Multiple Sequence Alignment and Phylogenetic Analysis

**Figure 1:**
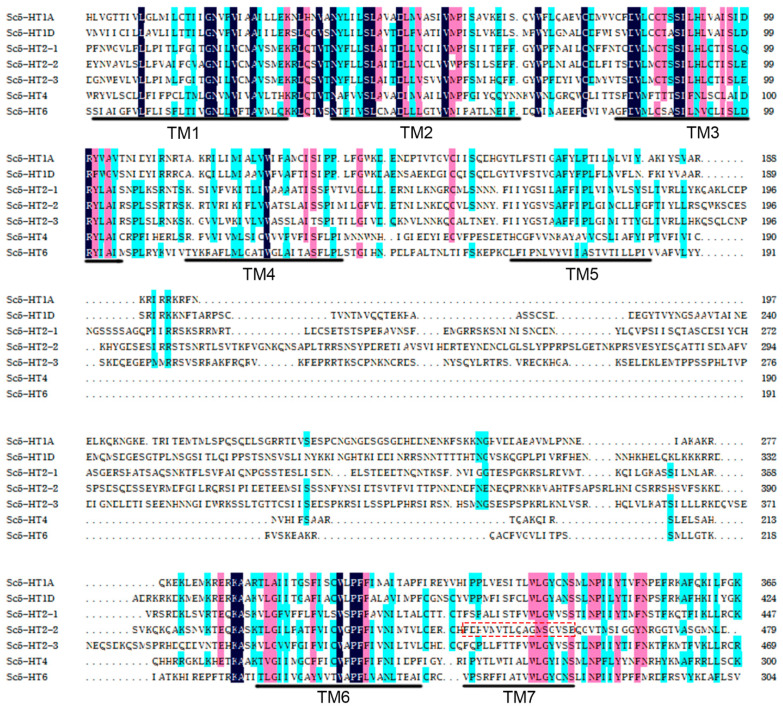
The multi-sequence alignment of the conserved domains of Sc5-HTRs (TM1, TM2, TM3, TM4, TM5, TM6, TM7: Transmembrance structures; Red dashed box represents partial protein sequence of Sc5-HT2-2 lack of TM7 structure. (The darker the color is, the higher the amino acid homology between sequences is. Three colors arranged by color depth represent 100% homology, ≥75% homology, and ≥50% homology).

The multi-sequence alignment revealed a 31.64% similarity between the *Sc5-HTRs*, with the concentrated regions primarily located within seven transmembrance structures, (TM1-M7) as depicted in Figure 1, which were consistent with the hallmark features of GPCRs. However, there were only six transmembrance structures (TM1-M6) in Sc5-HT2-2 compared with the other Sc5-HTRs (Table 3, Figure 1).

**Figure 2:**
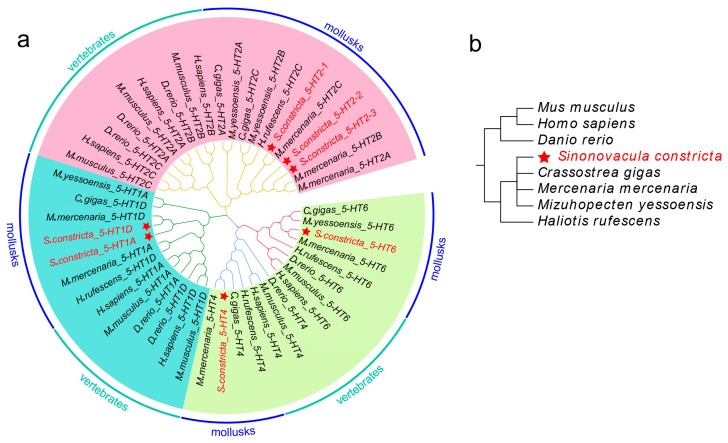
(**a**) Phylogenetic tree analysis of 5-HTRs constructed from *S. constricta* and other species with the maximum likelihood method by MEGA7 (3000 bootstrap replicates). The color of the branches represents the same receptor family, and 5-HT4 and 5-HT6 belong to the same family. The red star represents the 5-HT receptor of *S. constricta*. (**b**) The evolutionary trees of species was generated using the common tree function provided by the online platform NCBI.

To confirm the evolutionary and phylogenetic relationships, a maximum likelihood phylogenetic tree was constructed using 52 5-HTRs’ amino acid sequences from *H. sapiens*, *Mus musculus*, *Danio rerio*, *M. mercenaria*, Pacific oyster *Crassostrea gigas*, red abalone *Haliotis rufescens* and Yesso scallop *Mizuhopecten yessoensis* (Figure 2a). The phylogenetic relationships among the seven species are depicted in Figure 2b. The results showed that 5-HTRs could be classified into three clades: 5-HT1A and 5-HT1D were clustered together, while 5-HT4 and 5-HT6 formed another group, and 5-HT2A, 5-HT2B and 5-HT2C were clustered together. Overall, the Sc5-HTRs were initially grouped with mollusks but subsequently reclassified as vertebrates. 

### 3.3. Motif Patterns and Chromosome Location

**Figure 3:**
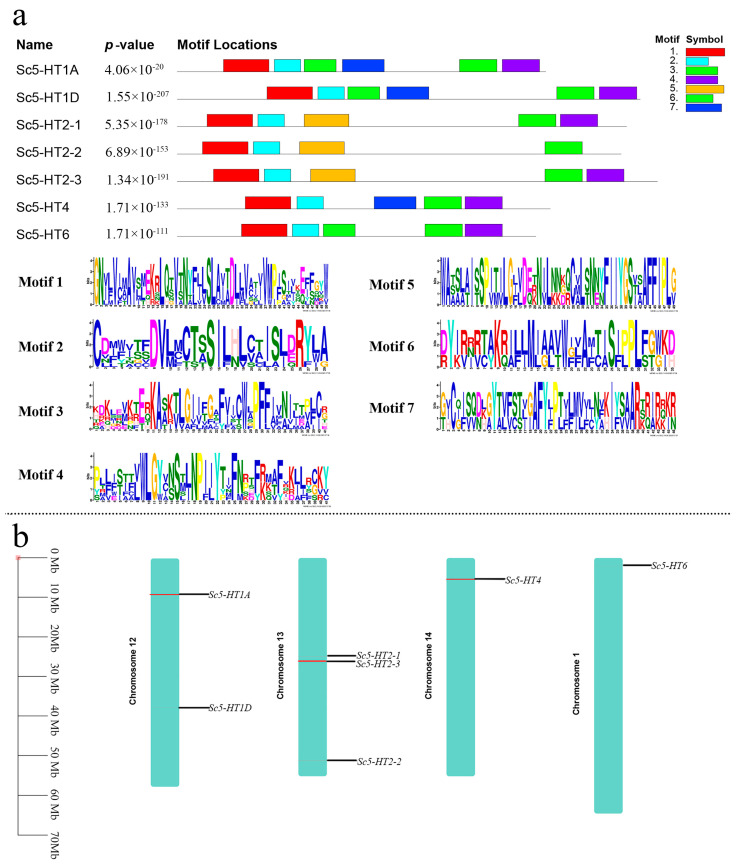
Motif and chromosome distribution analysis of *Sc5-HTRs*. (**a**) The distribution of conserved motif distributions and amino acid sequence of Sc5-HTRs; (**b**) The chromosome distribution analysis of *Sc5-HTRs*.

The results of motif analysis showed that seven conserved motifs were identified among the Sc5-HTRs (Figure 3a). Each Sc5-HTR protein contained motif 1, 2 and 3, and the same receptor family had similar motifs. Sc5-HT1A and Sc5-HT1D had the same motifs. In the Sc5-HT2 gene family, motif 4 was not found in Sc5-HT2-2, and motif 5 was specific to Sc5-HT2. Five motifs were identified in both Sc5-HT4 and Sc5-HT6, with the exception of motif 6 in Sc5-HT4 and motif 7 in Sc5-HT6. The presence of conserved motifs suggested homology between Sc5-HT4 and Sc5-HT6. The *Sc5-HTRs* were mapped on the *S. constricta* chromosomes (Figure 3b). The results showed that the seven *Sc5-HTRs* were distributed across four chromosomes, with the same gene families on the same chromosomes. Furthermore, both chromosome 1 (*Sc5-HT6*) and chromosome 14 (*Sc5-HT4*) exhibited an equal number of genes. Specifically, *Sc5-HT1* on chromosome 12 while *Sc5-HT2* was on chromosome 13.

### 3.4. Prediction of Tertiary Structure of Sc5-HTRs

**Figure 4:**
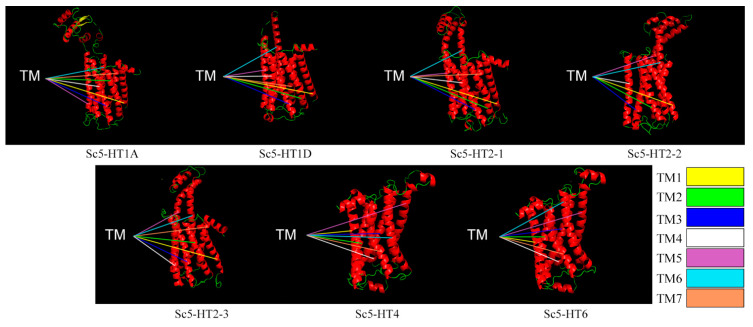
Potential tertiary structures of Sc5-HTR proteins (red represents helix, green represents loop, yellow represents sheet; the line color represents different TM structure).

The potential protein tertiary structures of the Sc5-HTRs were shown in Figure 4. Almost all of the Sc5-HTRs had a 5-HTR structure that was composed of three structural patterns: alpha helix, extended strand and random coil, and the numbers of amino acids of these three structures were different. The transmembrane structures (TM1~TM7) could be more intuitively visualized in the tertiary structure of Sc5-HTRs (Figure 4). 

### 3.5. Tissue Expression Pattern of Sc5-HTRs

**Figure 5:**
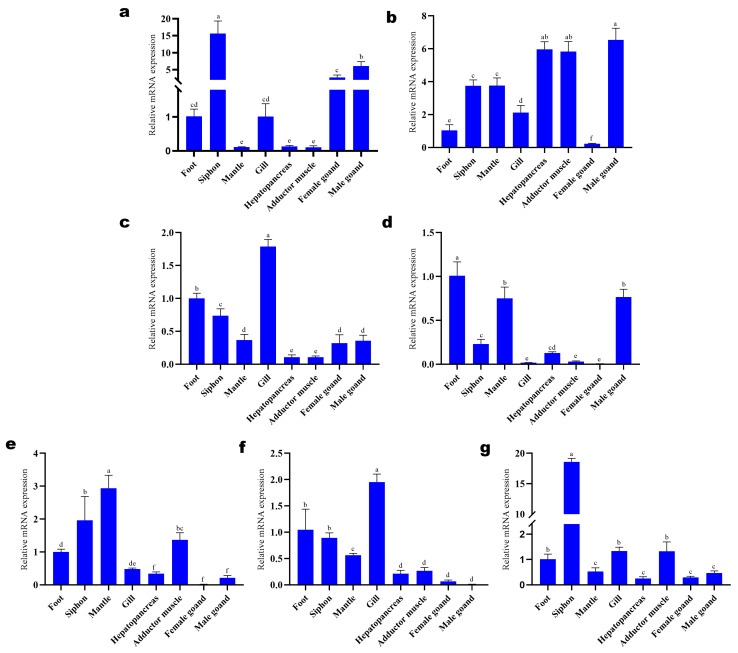
The mRNA expression levels of *Sc5-HTRs* in different tissues of *S. constricta* (n = 4). (**a**) *Sc5-HT1A.* (**b**) *Sc5-HT1D.* (**c**) *Sc5-HT2-1.* (**d**) *Sc5-HT2-2.* (**e**) *Sc5-HT2-3.* (**f**) *Sc5-HT4.* (**g**) *Sc5-HT6*. The method of 2^−ΔΔct^ was used to analyze the expression level of *Sc5-HTRs*, and the results were presented as mean ± S.E. The data were assessed using one-way ANOVA, and Student’s *t*-test was performed on each data set. Superscript letters represent statistically significant differences at *p* < 0.05.

The qRT-PCR results of *Sc5-HTRs* in different tissues revealed that the seven *Sc5-HTRs* were all expressed in eight tissues. However, the expression pattern of each *Sc5-HTR* had its own characteristics, and *Sc5-HTRs* in the same family were not similar (Figure 5). For example, *Sc5-HT1A* and *Sc5-HT6* were expressed highest in the siphon, which were about 10 folds higher than the other tissues. *Sc5-HT2-1* and *Sc5-HT4* were expressed highest in gills, while *Sc5-HT1D* was expressed higher in male gonads, the hepatopancreas and the adductor muscles. Whereas *Sc5-HT2-2* was mainly expressed in the foot, mantle and male gonad, the highest expression tissue of *Sc5-HT2-3* was found in mantle.

### 3.6. Preliminary Identification of Effective Sc5-HTRs That Binds to 5-HT

**Figure 6:**
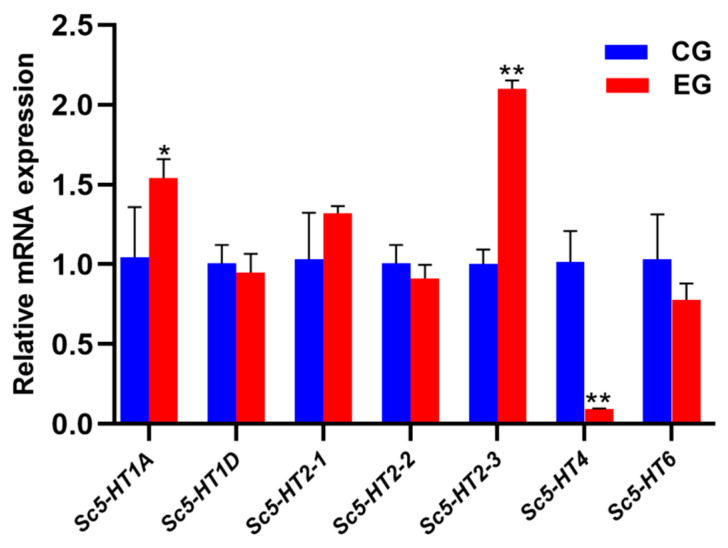
Expression changes of *Sc5-HTRs* after 5-HT injection (n = 4, CG: control group; EG: experimental group). The method of 2^−ΔΔct^ was used to analyze the expression level of *Sc5-HTRs*, and the results were presented as mean ± S.E. The data were assessed using one-way ANOVA, and Student’s *t*-test was performed on each data set (* represents *p* < 0.05, and ** represents *p* < 0.01).

Based on tissue expression results of *Sc5-HTRs*, high-expression tissues were selected to identify effective Sc5-HTRs that bind to 5-HT. After injecting 5-HT, the expression levels of *Sc5-HT1A*, *Sc5-HT2-3* and *Sc5-HT4* showed pharmacological reactions to 5-HT. The expression levels of *Sc5-HT1A* (*p* < 0.05) and *Sc5-HT2-3* (*p* < 0.01) increased significantly, whereas *Sc5-HT4* decreased significantly (*p* < 0.01) (Figure 6). 

### 3.7. Circadian Rhythm Expression Patterns of Sc5-HTRs

**Figure 7:**
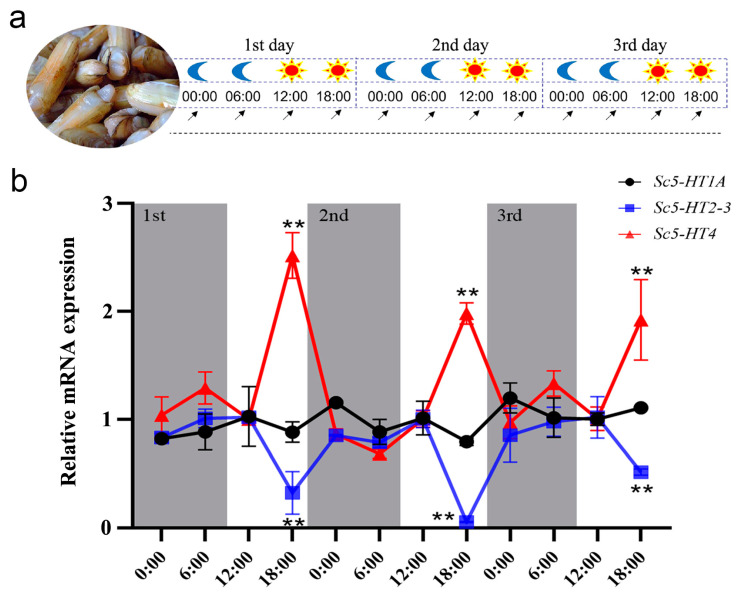
Circadian rhythm expression patterns of *Sc5-HTRs* within 72 h. (**a**) The experimental design of circadian rhythm. The moon represents the nighttime point, the sun represents the daytime point and the arrow represents the sampling time point. (**b**) Circadian rhythm expression patterns of *Sc5-HTRs* (n = 4). The results were presented as mean ± S.E, and Student’s *t*-test was performed on each data set (** *p* < 0.01).

Based on preliminary identification of effective Sc5-HTRs that bind to 5-HT, the circadian rhythm expression analysis was conducted for the *Sc5-HTRs* (Figure 7). The results showed that the expression level of *Sc5-HT1A* was stable at each time point, whereas *Sc5-HT2-3* and *Sc5-HT4* exhibited circadian rhythm expression patterns. *Sc5-HT2-3* expression was higher between 12:00 and 18:00 during the daytime and lower between 00:00 and 6:00 during the night. By contrast, the expression pattern of *Sc5-HT4* was opposite, with higher expression between 00:00 and 6:00 in the daytime and lower expression in the night. In general, the expression patterns of *Sc5-HT2-3* and *Sc5-HT4* showed circadian rhythms.

## 4. Discussion

Biogenic amines play an essential physiological function in mollusks [27,28,29]. As the largest family of receptors, GPCRs serve as the primary targets for biogenic amines such as 5-HT, dopamine, octopamine and acetylcholine [30]. 5-HT is an ancient neurotransmitter that binds to specific 5-HTRs in mollusks, which plays a crucial role in regulating spawning, parturition and meiosis reinitiation of prophase-arrested oocytes [18,19,20,21]. Thus, the study of gene structure and function of 5-HTRs have become increasingly significant. In the current study, seven *Sc5-HTRs* belonging to GPCRs were identified, and their relationships with nocturnal spawning were analyzed in *S. constricta*. 

Generally, elucidating the molecular structure will contribute to a better insight into the structure–function relationships of 5-HTRs. There are seven families of 5-HTRs, all of which are GPCRs except 5-HT3. In mollusks, several types of 5-HTRs have been cloned and identified. For instance, three 5-HTRs (5-HT1, 5-HT4 and 5-HT7) were identified in sea hare *Aplysia californica* [31], four 5-HTRs (5-HT1, 5-HT2, 5-HT3 and 5-HT4) have been identified in *C. gigas* [32] and so on. In the present study, seven Sc5-HTRs affiliated to four families (Sc5-HT1, Sc5-HT2, Sc5-HT4 and Sc5-HT6) were identified in *S. constricta*. The Sc5-HTRs exhibited a similarity of 31.64% with the conserved regions primarily located within six transmembrance structures (TM), which were characteristic homologous regions observed in other mollusks [33,34]. However, the Sc5-HT2-2 protein only possessed six TM regions that deviated from the typical structure of GPCRs. Truncated GPCRs have been shown to regulate the full-length version in both humans and abalones [34,35,36], suggesting a similar modulatory role for Sc5-HT2-2 on full-length 5-HTRs. Additionally, the predicted tertiary structure of the Sc5-HTRs conformed to the classical GPCRs, which demonstrated the reliability of identifying all seven receptors. 

Bioinformatics analysis is the fundamental approach for detecting the relationships among gene family members. Phylogenetic analysis and motif prediction showed that the similarity between Sc5-HT1 and Sc5-HT2 was low, while 5-HT4 and 5-HT6 were more closely related in terms of their phylogeny relationships and thus gathered together. Notably, all types of Sc5-HTRs display a greater degree of homology with mollusks, consistent with their evolutionary relationships. Compared to other receptors, 5-HT1 and 5-HT2 had a higher affinity for 5-HT [37]. The subdivision of 5-HT1 and 5-HT2 appears to have occurred after the divergence of vertebrates and invertebrates, resulting in divergent homology within mollusks [33]. The relatively low homology between *Sc5-HT1* and *Sc5-HT2* may be attributed to evolutionary and structural differences. Interestingly, Sc5-HT2 had more phosphorylation sites than other Sc5-HTRs, suggesting that the presence of multiple phosphorylation sites might lead to mediation of diverse functions via Sc5-HT2. Previous research has found that there were only three types of 5-HT2, yet it is involved in a multitude functions including aggressive behavior [38], promoting colon contraction [39] and food intake [40]. In our study, the high homology between *5-HT4* and *5-HT6* across various species meant that they could be classified as the same group, and there is evidence that 5-HT4 and 5-HT6 share similar executive functions in ancient mollusks and are undifferentiated [41]. For example, *5-HT4/6* was identified in the sea cucumber *Apostichopus japonicas*, which was undifferentiated [41]. As previously mentioned, these findings provided evidence that Sc5-HTRs had GPCRs characteristics and had higher homology with other 5-HTRs in mollusks, which were consistent with the results of evolution.

In mammals, 5-HTRs have tissue specificity to perform many functions, which made 5-HTRs derive unique tissue expression patterns [42]. The distribution of 5-HTRs had been reported in *Aplysia* [31,33], *C. gigas* [32] and abalone *H. discus hannai* [34]. 5-HT1 protein bands were detected in the gill, hermaphroditic and female and male gonads, as well as 5-HT2 protein in the bag cell of CNS, which may play a role in regulating afterdischarge during the spawning behavior in *A. californica* [31,33]. In *C. gigas*, *5-HTRs* were widely distributed in the hemolymph, gonad, mantle, gill, lip and hepatopancreas, and high expression levels in the mantle and hepatopancreas facilitated a more effective regulation of responses to air exposure [32]. Similarly, the predominant expressions of *Sc5-HT1* and *Sc5-HT2* were observed in the mantle, gill and hepatopancreas, which was consistent with *A. californica* and *C. gigas* [32,33]. In our study, the seven *Sc5-HTRs* were all expressed in the siphon, and exhibited a high level. Previous studies have found that 5-HT was scattered in the suprachiasmatic nucleus to adapt to change in photoperiodical variation [43], and the photosensitive cells were located in their siphons and occurred along the mantle margin in some mollusks [44,45], which suggested that *Sc5-HTRs* might be modulated by light to entrain the circadian rhythm of razor clams. In mollusks, 5-HT was distributed in ganglion and gonads, which was the same as the expression patterns of *5-HTRs*. *Sc5-HTRs* were expressed in male and female gonads, while the 5-HTRs present in crustaceans’ gonads could effectively stimulate the production of CHH/MIH/GIH hormones [46,47], indicating *Sc5-HTRs* might play important roles in gonad development.

5-HT is a neurotransmitter that exerts multiple effects through receptor binding and is commonly applied to induce mollusk spawning [48,49]. However, the precise role of 5-HT in reproductive processes remains unclear. Therefore, it was imperative to identify binding receptors for elucidating how 5-HT regulates spawning. In our study, effective *Sc5-HTRs* were investigated by 5-HT injection, and the expression levels of *Sc5-HT1A*, *Sc5-HT2-3* and *Sc5-HT4* were significantly altered, which suggested that *Sc5-HT1A*, *Sc5-HT2-3* and *Sc5-HT4* might be effective 5-HT receptors. The increased expression level of *Sc5-HT2-3* was observed to increase after the 5-HT injection, and similar results have been reported in *H. discus hannai* [16,50]. In the oocytes of mollusks, a transient increase of cytoplasmic Ca^2+^ concentration can regulate the key processes of fertilization and meiosis completion, even in a low Ca^2+^ water environment [51,52]. Only the second messenger IP3 of 5-HT2 had this function, which can cause a surge of intracellular Ca^2+^, suggesting *Sc5-HT2-3* might play similar functions in *S. constricta*. However, there is limited research on the function mechanism of other 5-HTRs in mollusks. It has been proved that 5-HT affects ovulation by regulating sex hormones in mollusks [49,53,54,55,56,57]. Moreover, sexual hormones could also exert an impact on the 5-HTR in mollusks, as evidenced by the downregulation of 5-HT4 after injection of 17β-estradiol in *H. discus hannai* [58]. This research may provide insights into mechanisms of 5-HT regulating reproduction in *S. constricta*, and will therefore need to be investigated in future studies.

It is commonly acknowledged that the majority of mollusks, including razor clams, exhibit a circadian rhythm and typically spawn during night [24]. 5-HT was located in the suprachiasmatic nucleus to regulate the circadian clock [43], and there was evidence that the 5-HT has seasonal and diel metabolic [59]. Studies have verified the existence of circadian rhythmicity of 5-HTR in crayfish [60]. In our study, the circadian rhythmic expression patterns of *Sc5-HTRs* were analyzed, and two distinct types of expression pattern were identified among the seven *5-HTRs*. The first type exhibited irregular expression patterns (*Sc5-HT1D*, *Sc5-HT2-1*, *Sc5-HT2-2* and *Sc5-HT6*), while the second type displayed regular expression patterns (*Sc5-HT1A*, *Sc5-HT2-3* and *Sc5-HT4*). These findings suggested that *Sc5-HTRs* exhibiting circadian rhythmic expression patterns might play important roles in regulating circadian behaviors. The expression of *Sc5-HT1A* remained stable during the day, without significant diurnal variation, which implied its non-involvement in the nocturnal spawning of *S. constricta*. Similarly, no diurnal rhythm changes of *5-HT1A* were found in the hippocampus measured in mice [61]. Interestingly, the expression levels of *Sc5-HT2-3* and *Sc5-HT4* underwent opposite circadian rhythmic changes. Existing research has demonstrated the critical role of regulating the biological functions of 5-HT2 and 5-HT4 in the suprachiasmatic nucleus [62,63]. Furthermore, it has been found that 5-HT2 could modulate circadian rhythm activities in mammals by affecting circuits involved in circadian behaviors [10,64]. Activation of 5-HT4 alters the expression levels of the circadian clock genes *Per* and *Bmal* [62]. Likewise, as effective 5-HTRs, the expression patterns of *Sc5-HT2-3* and *Sc5-HT4* exhibited circadian rhythmic changes, which proved that they might be involved in regulating nocturnal spawning in *S. constricta*. Moreover, it has been proved that 5-HT2 can directly affect intracellular Ca^2+^ dynamics to trigger spawning in marine bivalves [65], and further investigation is required to elucidate the roles of *Sc5-HT2-3* and Sc5-HT4 in the nocturnal spawning of *S. constricta*. 

## 5. Conclusions

Seven G protein receptors Sc5-HTRs (Sc5-HT1A, Sc5-HT1D, Sc5-HT2-1, Sc5-HT2-2, Sc5-HT2-3, Sc5-HT4 and Sc5-HT6) were identified from an *S. constricta* genome, which all conformed to the characteristics of the 5-HTR family. *Sc5-HT1A*, *Sc5-HT2-3* and *Sc5-HT4* were tentatively confirmed to be effective 5-HT receptors, and *Sc5-HT2-3* and *Sc5-HT4* were expressed in the circadian rhythm patterns by qRT-PCR, which were preliminarily relevant to the nocturnal spawning of *S. constricta*. Overall, three Sc5-HTRs were found to induce spawning by binding 5-HT in razor clams, suggesting that 5-HT could be utilized as a tool for further investigation into the mechanism of mollusk spawning.

## Figures and Tables

**Table 1:** The primer sequences of *Sc5-HTRs*.

Gene	GenBank Accession No.	Forward Primer (5′-3′)	Reverse Primer (5′-3′)
*Sc5-HT1A*	OR344072	GAACATCTAGTCGGCACCACCATC	GCCACAGCAAGTGAGAGGATAAGG
*Sc5-HT1D*	OR344075	TCAGACGGTGAAAGTGGGA	TGGTATGGGTTGTTGTGGTG
*Sc5-HT2-1*	OR344076	CAAACAGCGTCTTGCGATT	GAGATTCCGTTGATGAACCAG
*Sc5-HT2-2*	OR344077	GAACATCTAGTCGGCACCACCATC	GCCACAGCAAGTGAGAGGATAAGG
*Sc5-HT2-3*	OR344078	GAGGAGAATCACAACAATGGG	TACTGGTGGCTTTGAGAACAAG
*Sc5-HT4*	OR344073	CCGTTCATTGGATACAGGATTC	GCAACTAAGGAGCCGTCTGA
*Sc5-HT6*	OR344074	CATTCGGGAACCATTTACCA	CCGTCAAGTTTGCGACAAG
*RS9*	OQ244850	TGAAGTCTGGCGTGTCAAGT	CGTCCAAAAGGGCATTACC

**Table 2:** Similarity of *Sc5-HTRs* with corresponding proteins in other species.

Protein	Species	Similarity (%)	Accession Number
Sc5-HT1A	*Mercenaria mercenaria*	87.4	XP_045168822.1
Sc5-HT1D	*Mercenaria mercenaria*	72.6	XP_045169056.1
Sc5-HT2-1	*Mercenaria mercenaria*	74.1	XP_045200212.1
Sc5-HT2-2	*Mercenaria mercenaria*	58.2	XP_053404391.1
Sc5-HT2-3	*Mercenaria mercenaria*	78.1	XP_045200167.1
Sc5-HT4	*Mercenaria mercenaria*	82.7	XP_045189937.1
Sc5-HT6	*Mercenaria mercenaria*	77.9	XP_045159152.1

**Table 3:** Physicochemical properties of Sc5-HTRs.

Protein	Molecular Weight (kD)	Isoelectric Point (pI)	Transmembrane Structure	Phosphorylation Site
Sc5-HT1A	45.980	8.80	7	29
Sc5-HT1D	57.135	9.16	7	50
Sc5-HT2-1	54.932	9.40	7	66
Sc5-HT2-2	54.568	8.16	6	70
Sc5-HT2-3	60.478	9.34	7	67
Sc5-HT4	46.885	7.88	7	28
Sc5-HT6	43.945	9.19	7	33

## Data Availability

The datasets in this study can be found in online repositories. The names of the repository/repositories and accession number(s) can be found in the article.

## Supplementary Materials

The following supporting information can be downloaded at: https://www.mdpi.com/article/10.3390/ani13203208/s1, Table S1. Similarity of *Sc5-HTRs* with corresponding proteins in other species.
