# Supplementary material for: Genome-Wide Identification of 5-HT Receptor Gene Family in Razor Clam Sinonovacula constricta and Their Circadian Rhythm Expression Analysis
Source: Animals (Basel). 2023 Oct 14;13(20):3208. doi: 10.3390/ani13203208 (PMC10603676; doi:10.3390/ani13203208)
Supplement: Supplementary file 1 [file animals-13-03208-s001.zip › animals-2625459-supplementary.pdf]

Supplementary Table S1. Similarity of *Sc5-HTRs* with corresponding proteins in other species.

Protein	Species	Similarity (%)	Accession number
Sc5-HT1A	*Homo sapiens*	57.6	NP_000515.2
	Mus musculus	57.9	NP_032334.2
	Danio rerio	56.3	NP_001139238.1
	Crassostrea gigas	33.0	XP_011413902.2
	Mercenaria mercenaria	87.4	XP_045168822.1
	Mizuhopecten yessoensis	46.2	XP_021371618.1
Sc5-HT1D	*Homo sapiens*	46.0	NP_000855.1
	Mus musculus	45.6	NP_001272411.1
	Danio rerio	45.8	NP_001139158.1
	Crassostrea gigas	46.7	XP_011433775.2
	Haliotis rufescens	44.6	XP_053373023.1
	Mercenaria mercenaria	72.6	XP_045169056.1
Sc5-HT2-1	*Homo sapiens*	38.4	NP_000612.1
	Mus musculus	40.5	NP_766400.1
	Danio rerio	40.4	XP_009300470.1
	Crassostrea gigas	54.1	XP_011430856.2
	Haliotis rufescens	52.9	XP_046365275.1
	Mercenaria mercenaria	74.1	XP_045200212.1
	Mizuhopecten yessoensis	54.1	XP_021356300.1
Sc5-HT2-2	*Homo sapiens*	36.2	NP_001243689.2
	Mus musculus	35.0	NP_032338.3
	Danio rerio	31.0	NP_001038208.1
	Crassostrea gigas	32.8	XP_011430856.2
	Haliotis rufescens	35.2	XP_046365275.1
	Mercenaria mercenaria	58.2	XP_053404391.1
	Mizuhopecten yessoensis	31.2	XP_021356300.1
Sc5-HT2-3	*Homo sapiens*	41.3	NP_001307687.1

	Mus musculus	42.8	NP_032338.3
	Danio rerio	39.9	XP_009300470.1
	Crassostrea gigas	58.0	XP_011430856.2
	Haliotis rufescens	54.1	XP_046365275.1
	Mercenaria mercenaria	78.1	XP_045200167.1
	Mizuhopecten yessoensis	55.5	XP_021356300.1
Sc5-HT4	*Homo sapiens*	46.4	NP_001035263.1
	Mus musculus	51.4	NP_032339.2
	Danio rerio	50.8	XP_001337671.1
	Crassostrea gigas	67.1	XP_011430669
	Haliotis rufescens	64.1	XP_046371469.1
	Mercenaria mercenaria	82.7	XP_045189937.1
Sc5-HT6	*Homo sapiens*	39.3	NP_000862.1
	Mus musculus	38.3	NP_001364025.1
	Danio rerio	38.5	XP_009295353.1
	Crassostrea gigas	60.9	XP_011444450.2
	Haliotis rufescens	66.7	XP_046336019
	Mercenaria mercenaria	77.9	XP_045159152.1
	Mizuhopecten yessoensis	67.7	XP_021369720.1
